# Health Endpoint of Exposure to Criteria Air Pollutants in Ambient Air of on a Populated in Ahvaz City, Iran

**DOI:** 10.3389/fpubh.2022.869656

**Published:** 2022-03-29

**Authors:** Seyed Hamid Borsi, Gholamreza Goudarzi, Gholamreza Sarizadeh, Maryam Dastoorpoor, Sahar Geravandi, Habib Allah Shahriyari, Zahra Akhlagh Mohammadi, Mohammad Javad Mohammadi

**Affiliations:** ^1^Department of Internal Medicine, Air Pollution and Respiratory Diseases Research Center, Ahvaz Jundishapur University of Medical Sciences, Ahvaz, Iran; ^2^Air Pollution and Respiratory Diseases Research Center, Ahvaz Jundishapur University of Medical Sciences, Ahvaz, Iran; ^3^Department of Environmental Health Engineering and Environmental Technologies Research Center (ETRC), Ahvaz Jundishapur University of Medical Sciences, Ahvaz, Iran; ^4^Department of Biostatistics and Epidemiology, School of Health, Ahvaz Jundishapur University of Medical Sciences, Ahvaz, Iran; ^5^School of Public Health, Ahvaz Jundishapur University of Medical Sciences, Ahvaz, Iran; ^6^Department of Environmental Health Engineering, School of Public Health, Ahvaz Jundishapur University of Medical Sciences, Ahvaz, Iran

**Keywords:** air pollutants, health endpoint, cardiovascular mortality, respiratory disease, cardiovascular disease, Iran

## Abstract

The presence of criteria air pollutants (CAP) in the ambient air of a populated inhalation region is one of the main serious public health concerns. The present study evaluated the number of cardiovascular mortalities (CM), hospital admissions with cardiovascular disease (HACD), and hospital admissions for respiratory disease (HARD) due to CAP exposure between 2010 and 2014. The study used the Air Q model and descriptive analysis to investigate the health endpoint attributed to the ground level of ozone (O_3_), nitrogen dioxide (NO_2_), sulfide dioxide (SO_2_), and particle matter (PM_10_). Baseline incidence (BI) and relative risk (RR) are the most important factors in the evaluation of health outcomes from exposure to CAP in the ambient air of a populated area according to EPA and the World Health Organization (WHO) guidelines. Our study showed that annual cases of cardiovascular mortality during the period 2010–2014 relating to particle mater were 478, 506, 469, 427, and 371; ozone was 19, 24, 43, 56, and 49; nitrogen dioxide was 18, 20, 23, 27, and 21; and sulfide dioxide was 26, 31, 37, 43 and 11, in the years 2010 to 2014, respectively. These results indicate that the number of hospital admissions for respiratory disease attributed to PM were 2054, 2277, 2675, 2042, and 1895; O_3_ was 27, 35, 58, 73, and 63; NO_2_ was 23, 24, 15, 25, and 18; and SO_2_ was 23, 24, 25, 30, and 20, in the years from 2010 to 2014, respectively. The results also showed that the number of hospital admissions for cardiovascular disease related to particle mater was 560, 586, 529, 503, and 472; ozone was 22, 32, 38, 55, and 51; nitrogen dioxide was 19, 18, 13, 21, and 14; and sulfide dioxide was 12, 14, 16, 22, and 9, in the same period, respectively. Observations showed that most of the pollution was from outdoor air and in the human respiratory tract. Increased levels of sulfide dioxide, particle matter, nitrogen dioxide, and ozone can cause additional morbidity and mortality for exposed populations. According to the results, it is possible to help increase the level of public health. The use of these findings could also be of great help to health professionals and facilitators at regional and national levels.

## Highlights

- Ahvaz city is one of the most polluted cities in the Middle East and the world.
- PM_10_, NO_2_, SO_2_ and O_3_ significantly increased cardiovascular hospital admissions among human.
- Data shown here may motivate further studies would allow assessing the development in health status more precisely.
- Air pollution has had hazardous cardiovascular effects in Ahvaz city.

## Introduction

In recent years, the production and emission of air pollutants due to severe climate change, the uncontrolled increase of urbanization, increasing consumerism, high waste production, reducing rainfall, increasing desertification, as a result of the occurrence of dust phenomenon, the conversion of forest areas into agricultural fields, increasing pollution from industry and increasing emissions from transportation have been among the most serious problems. These factors cause heavy damage to human societies by increasing the number of hospitalizations and the cost of treatment, increasing the number of deaths, and intensification of economic and social damage caused by closures due to air pollution events (1, 2). The most important influencing factors are the type, size, and concentration of inhaled pollutants (3). Man-made pollutants are emitted from fixed sources (such as power plants, petrochemicals, chemical plants) and mobile sources (including transportation, cars, aircraft, trains, and growth of people) that increase the risk of disease in humans (4–6).

Respiratory diseases, pulmonary diseases, cardiovascular diseases, and deaths due to the high concentration of air pollutants and deeper penetration into the respiratory system are the main complications for human health (7, 8). In both developed and developing countries in recent decades, air pollution (ground level of ozone, nitrogen dioxide, sulfide dioxide, and particle matter) has become one of the most important factors that cause many health effects such as asthma, bronchitis, respiratory and cardiovascular disease, and different cancers (stomach, blood, liver, lung, kidney, brain) and death (7). Based on a report by the World Health Organization (WHO) and another health organization, ~6.4 million people have Disability-Adjusted Life Year (DALY) and 7 million people have died due to exposure to pollutants in indoor and outdoor air (9–11). The main factors that determine the severity of health consequences depend on the concentration of pollutants in ambient air and the duration of exposure to polluted air (12, 13). The most important way air pollutants enter the body are through the respiratory system, digestion, swallowing, skin, nasal mucosa, and eyes, which cause various diseases including cataracts, skin diseases (itching, redness, and dry skin), cardiovascular disease, the upper and lower respiratory system, gastrointestinal diseases, various cancers (stomach, intestines, brain, lungs, ovaries, and prostate), infertility and unwanted abortion, especially in groups sensitive to air pollution (children, the elderly, heart and respiratory patients) and eventually death (4, 14–16).

The concentration and number of pollutants are influenced by several parameters such as aerosol composition, aerosol charge, particle dryness, division power, wind velocity, and sampling volume (17). Exposure to air pollutants during exercise has more negative effects on lung health due to higher exposure to pollutants. Decreasing the use of fossil fuels, modifying the industrial production process, improving the fuel quality of conveyors and increasing public transportation can be effective in diminishing the detrimental effects on the economy and health because of breathing in air pollutants. If the methods are followed properly and seriously, it is possible to prevent the inhalation of polluted air, which can cause a lot of destructive and irreparable damage to the environment, animals, and humans (18). Extreme exercise increases the number of breaths per minute. In many cases, athletes breathe through the mouth instead of the nose when exercising, meaning that many air pollutants such as bioaerosols can easily penetrate the lungs (19).

The main risks that threaten the environment, animal, and human health include rapid population growth, wastewater, climate change, dust storms, and the development of industries such as petroleum, gas, oil, and steel in regions of Iran (20). This study aimed to investigate the consequences of exposure to ozone, nitrogen dioxide, particle matter, and sulfide dioxide and evaluate cardiovascular mortality, hospital admission respiratory disease, and hospital admission cardiovascular disease in Ahvaz megacity from 2010 to 2014.

## Materials and Methods

### Study Design

This is a cross-sectional study undertaken in Ahvaz megacity. In this study, the number of deaths (per 100,000 population) includes all-cause mortality cardiovascular in people, HACD in residents of Ahvaz, and HARD in adults attributable to exposure to PM_10_, O_3_, NO_2_, and SO_2_ in Ahvaz will be assessed over the period (2010–2014). Table 1 shows the number of populations at risk for health effect assessment of CAP.

**Table 1:** Criteria air pollutants concentrations (μg/m^3^) and Health effects assessment of CAP on Ahvaz megacity inhabitants, 2010-2014.

**Health effects**	**Air pollutants**	**RR (Health effects)**	**Annual mean**	**Cardiovascular mortality**	**HARD**	**HACD**
2010	Particle mater	CM = 1.008 (1.005–1.018) HARD = 1.0080 (1.0048–1.0112) HACD =1.009 (1.006-1.013)	281.98	478	2,054	560
2011	Particle mater		288.38	506	2,277	586
2012	Particle mater		278.12	469	2,675	529
2013	Particle mater		242.29	427	2,042	503
2014	Particle mater		205.52	371	1,895	472
2010	Sulfide dioxide	CM = 1.008 (1.005–1.018) HARD = 1.0044 (1–1.011) HACD = 1.0064 (1.0026–1.0101)	78.92	26	23	12
2011	Sulfide dioxide		91.07	31	24	14
2012	Sulfide dioxide		92.75	37	25	16
2013	Sulfide dioxide		112.3	43	30	22
2014	Sulfide dioxide		88.57	11	20	9
2010	Ozone	CM = 1.0080 (1.004–1.012) HARD = 1.0040 (1.002–1.006) HACD =1.0058 (1.0022−1.0094)	66.52	19	27	22
2011	Ozone		72.67	24	35	32
2012	Ozone		102.27	43	58	38
2013	Ozone		223	56	73	55
2014	Ozone		187	49	63	51
2010	Nitrogen dioxide	CM = 1.002 (1–1.004) HARD = 1.0038 (1.0004–1.0094) HACD = 1.009 (1.006–1.013)	28.7	18	23	19
2011	Nitrogen dioxide		31	20	24	18
2012	Nitrogen dioxide		37	23	15	13
2013	Nitrogen dioxide		41	27	25	21
2014	Nitrogen dioxide		34	21	18	14

*CM, Cardiovascular Mortality; HARD, Hospital Admission Respiratory Disease; HACD, Hospital Admission Cardiovascular Disease. RR, is a ratio of the probability of the event occurring in the exposed group vs. a non-exposed group. AP, is defined as the part of health effects which can be attributed to the pollutant exposure in population. In preparing Table 1, which includes the concentration of pollutants and their effects, the mentioned studies have been used (21–25)*.

**Figure 1:**
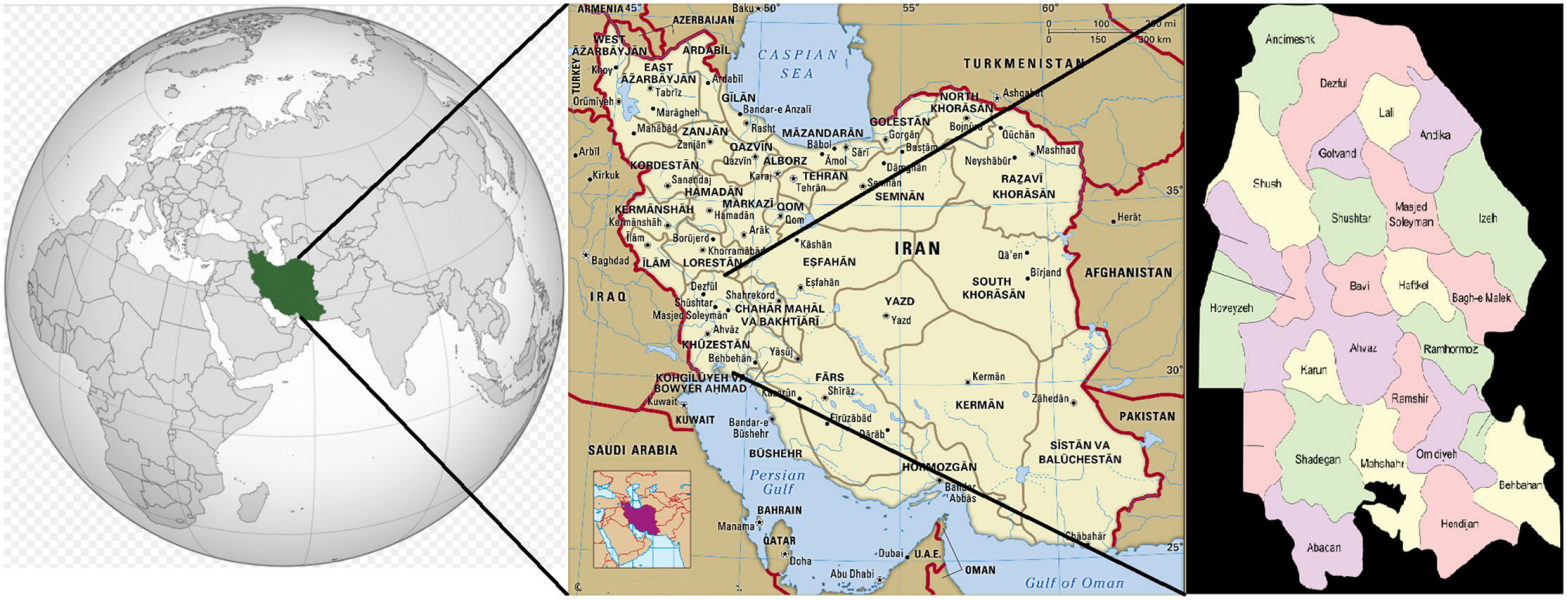
Located of sampling stations in Ahvaz megacity.

Ahvaz with an area of 185 km^2^ at the center of Khuzestan province, located in the southwest of Iran and to the north of the Persian Gulf, located at 31° 19′ 13″ N, 48° 40′ 9″ E. Ahvaz is one of the largest cities in Iran (26). The population of Ahvaz is 1,300,000. The climate is warm most of the time, with warm summers and long and moderate winters, and temperatures reaching as high as 50°C. The land area is 140 km^2^ (27). PM_10_, O_3_, NO_2_, and SO_2_ data from the Department of Environment and Meteorological Organization were collected from the Deputy of Health (Ahvaz city). Based on these criteria, four monitoring stations were selected. The geographical location of the area under study (Ahvaz city) is shown in Figure 1.

### Air Q Software

Quantification of PM_10_, O_3_, NO_2_, and SO_2_ pollutants for health impact assessment (HIA) was performed by employing Air Q modeling software.

Air Q software consists of two models of quantification and life tables. These life tables and health effects were used in this study. Population contact data including demographics, health outcomes, and air quality data were fed into the software. The relative risk (RR) in formula 1 was used to calculate the Attributable proportion (AP) value (4, 27–29).


(1) $$AP\;=\;\sum^{\text{​}}([RR(c)-1]*P(c)])/\sum^{\text{​}}[RR(c)*P(c)]$$

In which AP is equal to the proportion of the population exposed to the pollutant over a specified period of time (attributed ratio), RR (c) is the relative risk of health effects on the target population in the contact group c and P (c) equals the population ratio of the exposure group c.

The number of cases per 100,000 population at risk (BE) can be calculated by Formula 2.


(2) $$\begin{array}{r} \text{BE}=\text{B }\times \text{ AP} \end{array}$$

B represents the number of health outcomes per 100,000 populations at risk. Due to the inaccessibility of hospital data, baseline incidence (BI) values were used from other studies (28, 30, 31). AP can calculate through the following formula (32):


(3) $$\begin{array}{r} IE\;=\;I\times \;AP \end{array}$$

The total number of cases attributable (NE) was calculated using Formula 4 to evaluate the health effects on the number of at-risk populations (N) (shown in Table 1).


(4) $$\begin{array}{r} NE\;=IE\times \;N \end{array}$$

### Data Collection

The present study is an epidemiological study that correlated the concentration of PM_10_, O_3_, NO_2_, and SO_2_ to the number of CM, HARD, and HACD during the period 2010-2014. The hourly concentrations of CAP were obtained from the Department of Environment (DOE) Ahvaz.

Classification of health endpoint in this study is based on the International Classification of Diseases (ICD-10) indication for total mortality (TM- J95.150), hospital admission respiratory disease (HARD- J44.8), hospital admission cardiovascular disease (HACD- I51.6), and cardiovascular mortality (CM- I25.8) (33–35). The health effect in this study was investigated with used baseline incidence, relative risk, and proportion attributed to CAP by the Air-Q model.

## Results

Investigation of cardiovascular mortality, hospital admission cardiovascular disease, hospital admission respiratory disease and the relationship between criteria air pollutants and needs to gather information exposed population and concentration of O_3_, PM_10_, SO_2_, and NO_2_.

Table 1 shows the number of health endpoints related to the criteria air pollutant concentrations on Ahvaz citizens during 2010-2014. It shows that the trend of yearly concentration of particle matter was decreased and related to ozone and that nitrogen dioxide and sulfide dioxide increased during 2010-2014.

As shown in Table 1, the level of sulfide dioxide, nitrogen dioxide, and ozone in 2013 was at a maximum. In this study central relative risk according to sulfide dioxide, particle matter, nitrogen dioxide, and ozone were used to calculate the number of cases (Table 1).

Based on the result, the annual level of CAP during the same period were SO_2:_ 78.92, 91.07, 92.75, 112.3, and 88.57 μg/m^3^; NO_2:_ 28.7, 31, 37, 41, and 34 μg/m^3^; PM_10:_ 281.98, 288.38, 278.12, 242.29, and 205.52 μg/m^3^; O_3:_ 66.52, 72.67, 102.27, 223, and 187 μg/m^3^, respectively.

Finding showed that, the number of CM attributed to particle mater were (478, 506, 469, 427, and 371); ozone (19, 24, 43, 56, and 49); nitrogen dioxide (18, 20, 23, 27, and 21); and sulfide dioxide (26, 31, 37, 43, and 11), in 2010 until 2014, respectively (Table 1). The number of HARD attributed to PM were (2,054, 2,277, 2,675, 2,042, and 1,895); O_3_ (27, 35, 58, 73, and 63); NO_2_ (23, 24, 15, 25, and 18); and SO_2_ (23, 24, 25, 30, and 20), during 2010-2014, respectively (Table 1).

Table 1 shows that the number of HACD related to particle mater were (560, 586, 529, 503, and 472); ozone (22, 32, 38, 55, and 51); nitrogen dioxide (19, 18, 13, 21, and 14); and sulfide dioxide (12, 14, 16, 22, and 9), in 2010 until 2014, respectively.

**Figure 2:**
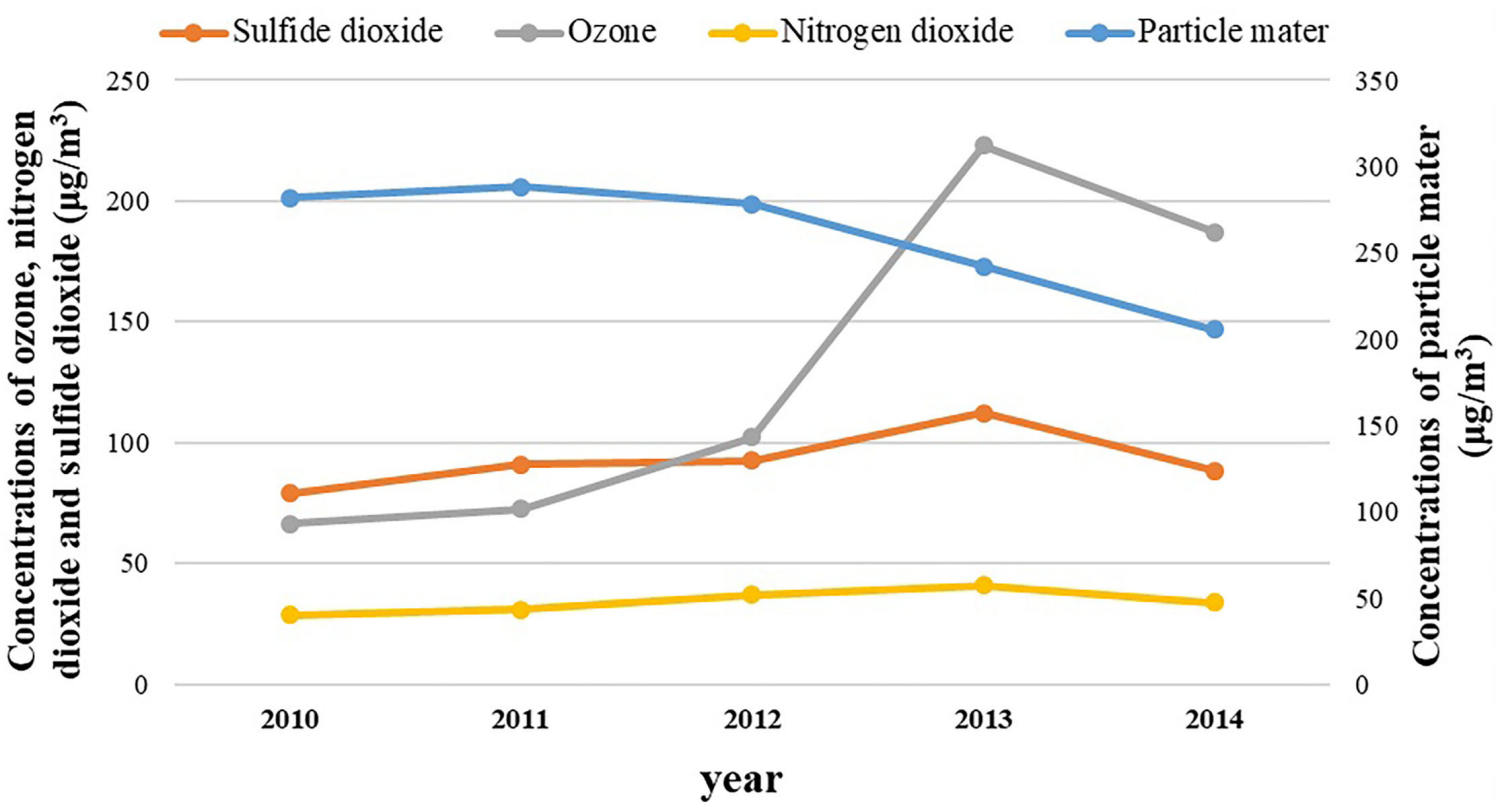
Annual averages of the criteria air pollutants during the study period in ambient air of Ahvaz City, Iran.

Figure 2 presents the annual average of the criteria air pollutants in ambient air of Ahvaz City, Iran. As depicted in Figure 2, particle matter was a decreasing trend and other pollutants including ozone, sulfide dioxide and nitrogen dioxide had an increasing and then a decreasing trend.

**Figure 3:**
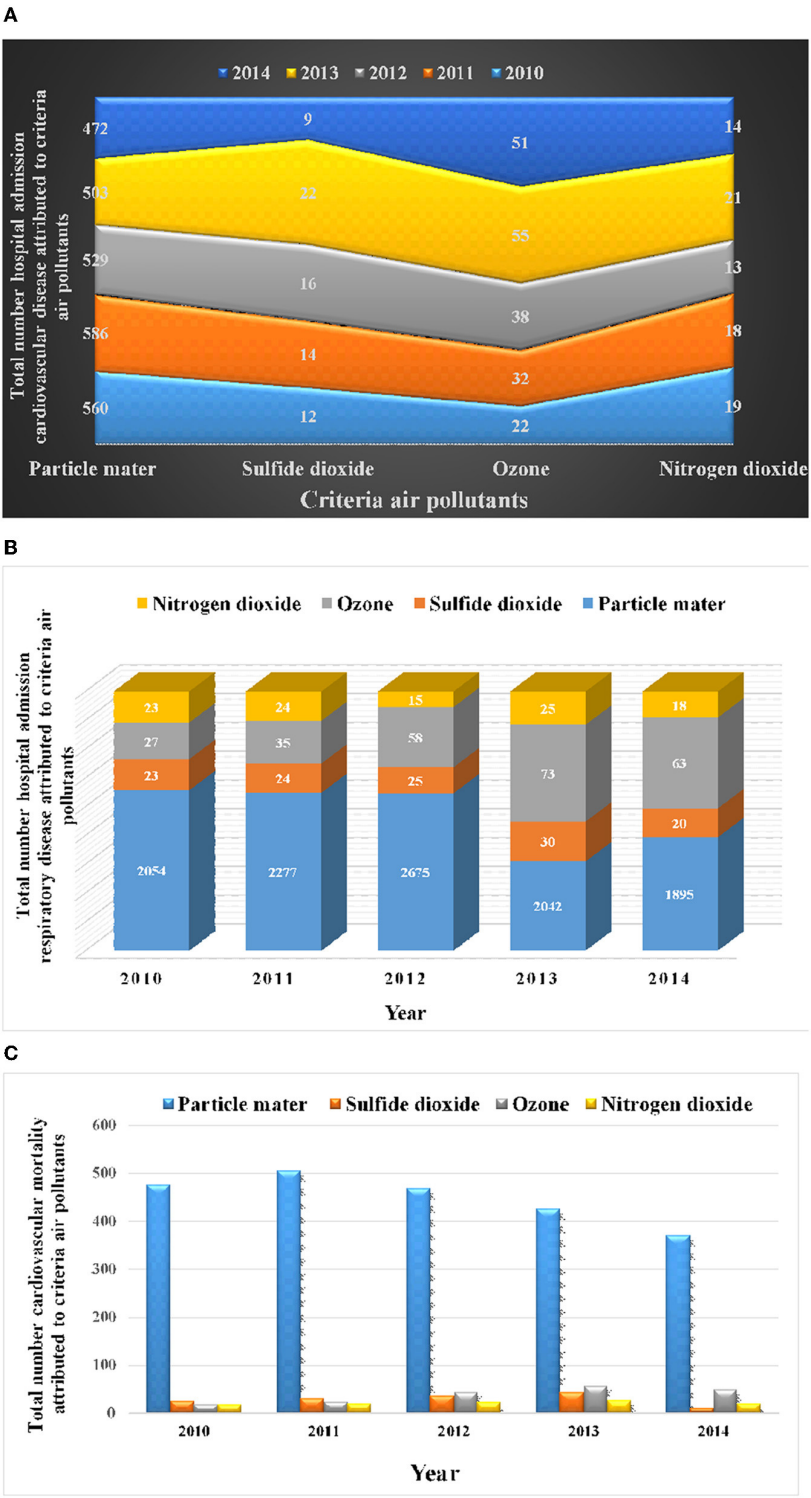
Cumulative number of cardiovascular mortality, hospital admission respiratory disease, hospital admission cardiovascular disease related to criteria air pollutants. **(A)** Number of hospital admission cardiovascular disease. **(B)** Number of hospital admission respiratory disease. **(C)** Number of cardiovascular mortality.

The cumulative number of cardiovascular mortality, hospital admission respiratory disease, hospital admission cardiovascular disease related to criteria air pollutants during 2010 to 2014 is illustrated in Figure 3. Figure 3 shows that the number of health endpoints of exposure to criteria air pollutants were characterized by a sinusoidal process (first increasing and then decreasing).

## Discussion

Recently the disadvantages of ozone, nitrogen dioxide, particle matter, and sulfide dioxide on physical and mental health have attracted the attention of many researchers. According to the result of this study most cases of CM, HACD and HARD were related to O_3_, PM_10_, SO_2_, and NO_2_ during the year 2013. The results also showed that the highest calculated number of cases health endpoint regarding PM_10_ was from 2010 to 2011.

Based on these findings, the annual level of average ozone, nitrogen dioxide, particle mater and sulfide dioxide concentrations during the same period were 66.52, 72.67, 102.27, 223, and 187 μg/m^3^; 28.7, 31, 37, 41, and 34 μg/m^3^; 281.98, 288.38, 278.12, 242.29, and 205.52 μg/m^3^; 78.92, 91.07, 92.75, 112.3, and 88.57 μg/m^3^, respectively.

These results indicated that the number of CM attributed to particle mater were (478, 506, 469, 427, and 371); ozone (19, 24, 43, 56, and 49); nitrogen dioxide (18, 20, 23, 27 and 21); and sulfide dioxide (26, 31, 37, 43, and 11), in the years 2010 to 2014, respectively. The number of HACD related to particle mater were (560, 586, 529, 503, and 472); ozone (22, 32, 38, 55, and 51); nitrogen dioxide (19, 18, 13, 21, and 14); and sulfide dioxide (12, 14, 16, 22, and 9), in 2010 until 2014, respectively. The number of HARD attributed to PM were (2054, 2277, 2675, 2042, and 1895); O_3_ (27, 35, 58, 73 and 63); NO_2_ (23, 24, 15, 25, and 18); and SO_2_ (23, 24, 25, 30, and 20), during 2010-2014, respectively.

Table 1 shows the trends of level nitrogen dioxide, sulfide dioxide, ozone, and particle matter and the health endpoint of exposure to criteria air pollutants in ambient air of on a populated in Ahvaz city, Iran.

Also, the result specified that the mean concentration of air pollutants was higher than WHO guidelines standard. As can be seen in Figure 2, cases of cardiovascular mortality, hospital admission respiratory disease, and hospital admission cardiovascular disease related to PM_10_, SO_2_, NO_2_, and O_3_ in the period of the study had an increasing and then a decreasing trend.

Yao et al. (36) undertook a China evaluation of health burden related to exposure to air pollutants. They reported that exposure to air pollutants cause cardiovascular and respiratory disease in 7.8 million people (36). They reported that the premature mortality, contributions of PM_2.5_, PM_10_, NO_2_, SO_2_, O_3_, and CO were11.1, 5.2, 28.9, 9.6, 23.0, and 22.2%, respectively (36).

In 2013, Jeong undertook a study of Suwon, Korea, which examined the health effects of air pollution on people. The study observed that the annual PM10 average was equal to 52 μg/m^3^. The results of the study suggest that the summer season with a concentration of 2,563 μg/m^3^ was the highest amount of PM_10_ μg/m^3^ (37).

In Egypt Shakour et al. reported that for every 10 μg/m^3^ in PM10 concentration, there would be a 4.1% increase in hospital admission cardiovascular disease (HARD) (38), which is lower compared to other studies. A 2013 study by Habeebullah et al. (39) in Makkah, Saudi Arabia found that the annual average and maximum PM10 were 195.5 and 782.1 μg/m^3^, which were lower than the levels of PM10 in our study. Particle concentrations in the present study also had the highest concentrations in summer, which is the main reason this increase could be due to the occurrence of dust storms in the Middle East areas because of wind speed and higher temperature.

DeFlorio-Barker et al. (40) estimated the air pollution levels and population influence interaction of exposure to air pollution. They showed that exercise intensifies the ill effects of air pollution for those with chronic conditions (40).

Kumarathasan et al. (41) in the vicinity of a steel mill assessment of healthy humans between cardiovascular and inflammatory mechanisms and exposure to air pollution in healthy humans. The results showed that the mean CO, SO_2_ and ultrafine particle (UFP) levels on the day of biological sampling were higher at the Bayview site compared to the College site (41). They reported that the steel mill site can influence inflammatory and vascular mechanisms (41).

In studies performed by Yarahmadi et al. (42) in Tehran on mortality related to exposure to fine particles, the results showed that mortality was related to a corresponding reduction in PM concentrations. The annual mean cases of chronic obstructive pulmonary disease and lung cancer deaths related to exposure to PM_2.5_ were 158 and 142, respectively (42).

The results of this study showed that the level of pollutants in Ahvaz was a great deal higher compared to standards outlined in heavy industries such as oil, gas, petrochemical, and steel. The reasons for this might include dust storms, a decrease in rainfall, and an increase in drought.

Biggeri et al. (43) studied the relationship between the health effects attributed to long-term exposure to sulfur dioxide and health endpoint on citizens in Italy. This study reported a significant relationship between health effects and SO_2_ (43). The result of this study showed that an increase of 10 μg/m^3^ in sulfur dioxide was associated with an increase of 2.4% in hospital admissions (43).

High emissions of high sulfur from heavy industries could be related to the high percentage of hospital admissions for respiratory and cardiovascular disease in Ahvaz.

Bell et al. (44) in the U.S undertook similar work, in 2004, they studied the relationship between health effects and ground-level ozone. Results showed that a high level of ground-level ozone increased the risk of daily deaths, hospital admissions, and respiratory and cardiovascular diseases (10-ppb increase in the concentration of ground-level ozone was attributed to a 0.52% increase in daily deaths) (44). The most important reasons that can be mentioned for the difference between the results of this study and other studies is the existence of climatic characteristics, geography and meteorological changes in the city of Ahvaz.

Samoli et al. (45) conducted a study in 30 European cities, outlining that there was a relation between NO_2_ levels and cardiovascular and respiratory mortality. According to their results, a significant association was found between cardiovascular and respiratory mortality and the level of nitrogen dioxide (45). They indicate that cities with a larger proportion of elderly persons in the population were observed to have a higher number of respiratory mortality (45).

In 2016, Dijkema et al. (46) studied the relation between cardiopulmonary hospital admissions and variation in concentrations of nitrogen dioxide. They found that there is a higher risk of cardiopulmonary hospital admissions in areas with a higher concentration of nitrogen dioxide (46). Findings showed that positive associations were found between the levels of nitrogen dioxide and hospital admission rates for asthma, chronic obstructive pulmonary disease (COPD), all cardiovascular causes, ischemic heart disease, and for the second to fourth quartile relative to the first quartile of exposure were 1.87 (1.46–2.40), 2.34 (1.83–3.01), and 2.81 (2.16–3.65) for asthma; 1.44 (1.19–1.74), 1.50 (1.24–1.82), and 1.60 (1.31–1.96) for COPD) (46).

The city of Ahvaz has also been significantly affected by rapid population growth, heavy industry, lack of proper transportation, and dust storms. Since air is the main factor in the life of humans, breathing large amounts of pollutants can pose health risks to the people in the region. Activities related to the development of oil and gas fields and petrochemical and steel project industries and pipelines have increased the concentration of other pollutants. Therefore, continuous monitoring of the air pollutant content in indoor and outdoor air quality is a priority. The trend of yearly concentration of air pollutants was decreasing and showed that the action taken to reduce the emission of these contaminants through corrective actions such as improving the quality of fuel in vehicles, increasing the planting of trees, improving the quality of services provided in the public transport sector, reducing the production and emission by industries are effective.

## Limitations and Strengths

The main limitations of this study were a lack of epidemiological studies and calculations of relative risk (RR) and baseline incidence (BI) attributed to cardiovascular mortality, hospital admission respiratory and cardiovascular disease in humans due to criteria air pollutants in the study area. To solve this problem, it is necessary to conduct cohort studies for several years to calculate RR and BI. In recent years, a study of the Hoveyzeh cohort has started in the southwest of Iran. It is hoped that in a few years, important indicators such as baseline incidence and relative risk in this region will be calculated and this problem will be solved in future studies.

The most important strengths of the study were the investigation of the health endpoint of exposure to criteria air pollutants in ambient air for this area (Ahvaz City, Iran). Estimating the risks of air pollutants on human health can play an important role in raising public awareness. They can also help health policymakers by providing a practical guide for policy. In future studies, examination of time series analysis will be done with the help of statistics experts.

## Conclusion

We evaluated the consequences of exposure to particle matter (PM_10_), ground level of ozone (O_3_), sulfide dioxide (SO_2_), and nitrogen dioxide (NO_2_) in the ambient air of a populated area in Ahvaz City, Iran. Considering that Ahvaz is one of the largest cities in Iran and the region from the aspect of urbanization, industry, and population, these findings could be useful for decision-makers in other parts of the country and the world, especially in the Middle East. According to the findings, there is a significant relationship between cardiovascular mortality (CM), hospital admissions cardiovascular disease (HACD), hospital admission respiratory disease (HARD), and increased exposure to air pollutants.

The results of this study showed that the concentration of PM_10_, O_3_, SO_2_, and NO_2_ was higher than the WHO guideline value. The main agents of cases health effects of exposure to PM_10_ were because of the high level of this pollutant. Paying attention to decreasing emissions and levels of particle matter, the ground level of ozone, sulfide dioxide, and nitrogen dioxide for the reduction of cases of CM, HACD, and HARD attributed to particle matter, nitrogen dioxide, sulfide dioxide, and ozone b are important.

Observing modern urban planning patterns in urban development, developing public transportation (especially metro), increasing green space per capita by planting trees and creating parks in different areas of the city, carrying out effective activities in desertification, car replacement (worn and old), creating green belts around cities, reducing emissions due to the activities of heavy industries (oil and gas), improving production processes in important industries (steel, refineries, and cement), the establishment of monitoring and measuring stations, careful monitoring and increasing the general literacy of society are the most important control activities that will reduce the concentration of pollutants and harmful effects of inhaling these substances.

## Data Availability Statement

The original contributions presented in the study are included in the article/supplementary material, further inquiries can be directed to the corresponding author.

## Author Contributions

SB, GG, GS, MD, SG, HS, ZA, and MM: study concept, design, and critical revision of the manuscript for important intellectual content. MM: drafting of the manuscript and advisor. MD, SG, HS, and ZA: performing the experiments. SB, GG, MM, GS, and MD: revised the manuscript and finalizing. All authors contributed to the article and approved the submitted version.

## Funding

This study was funded by the Air Pollution and Respiratory Diseases Research Center of Ahvaz Jundishapur University of Medical Sciences (APRD-9906). The authors are grateful to the Department of Environment of Ahvaz city for providing data as well as the Air Pollution and Respiratory Diseases Research Center of Ahvaz Jundishapur University of Medical Sciences (with code IR.AJUMS.REC.1399.444) for providing the necessary facilities to perform this research.

## Conflict of Interest

The authors declare that the research was conducted in the absence of any commercial or financial relationships that could be construed as a potential conflict of interest.

## Publisher's Note

All claims expressed in this article are solely those of the authors and do not necessarily represent those of their affiliated organizations, or those of the publisher, the editors and the reviewers. Any product that may be evaluated in this article, or claim that may be made by its manufacturer, is not guaranteed or endorsed by the publisher.
